# Cytoreduction surgery reduces systemic myeloid suppressor cell populations and restores intratumoral immunotherapy effectiveness

**DOI:** 10.1186/1756-8722-5-34

**Published:** 2012-06-28

**Authors:** Jarrod D Predina, Veena Kapoor, Brendan F Judy, Guanjun Cheng, Zvi Gregory Fridlender, Steven M Albelda, Sunil Singhal

**Affiliations:** 1 Department of Surgery, Thoracic Surgery Research Laboratory, University of Pennsylvania School of Medicine, Philadelphia, PA, USA; 2 Department of Medicine, Division of Pulmonary, Allergy and Critical Care, University of Pennsylvania School of Medicine, Philadelphia, PA, USA; 3 University of Pennsylvania School of Medicine, 6 White Building, 3400 Spruce Street, Philadelphia, PA, 19104, USA

**Keywords:** Surgical oncology, Immunotherapy, Cancer, Animal model

## Abstract

**Background:**

Multiple immunotherapy approaches have improved adaptive anti-tumor immune responses in patients with early stage disease; however, results have been less dramatic when treating patients with late stage disease. These blunted responses are likely due to a host of factors, including changes in the tumor microenvironment and systemic immunosuppressive features, which accompany advanced tumor states. We hypothesized that cytoreductive surgery could control these immunosuppressive networks and restore the potency of immunotherapy in advanced disease scenarios.

**Methods:**

To test these hypotheses, two representative intratumoral immunotherapies (an adenoviral vector encoding a suicide gene, AdV-tk, or a type-I interferon, Ad.IFNα) were tested in murine models of lung cancer. Cytoreductive surgery was performed following treatment of advanced tumors. Mechanistic underpinnings were investigated using flow cytometry, *in vivo* leukocyte depletion methods and *in vivo* tumor neutralization assays.

**Results:**

AdV-tk and Ad.IFNα were effective in treating early lung cancers, but had little anti-tumor effects in late stage cancers. Interestingly, in late stage scenarios, surgical cytoreduction unmasked the anti-tumor potency of both immunotherapeutic approaches. Immune mechanisms that explained restoration in anti-tumor immune responses included increased CD8 T-cell trafficking and reduced myeloid derived suppressor cell populations.

**Conclusion:**

This study demonstrates that surgical resection combined with immunotherapy may be a rational therapeutic option for patients with advanced stage cancer.

## Background

A number of intratumoral immunotherapies are currently being examined in clinical trials [1,2]. In these trials, limited success has been observed in patients with advanced tumor burdens [3]. Potential reasons for the lack of effectiveness of intratumoral immunotherapy in advanced cancer states include the development of complex immunosuppressive features: (i) low CD8 T-cell to tumor ratio [4], (ii) extensive systemic suppressive cell populations such as myeloid derived suppressor cells (MDSCs) [5] and T-regulatory cells [6], (iii) poor T-cell trafficking into the tumor [7], (iv) an immunosuppressive tumor microenvironment [8], and (vi) increased levels of soluble immunosuppressive substances such as prostaglandin E2 [9], TGF-β [10], VEGF [11] or IL-10 [12,13].

Interestingly, several years ago our group made the observation that immunotherapy (in the form of Ad.IFNβ) was effective in treating very large mesothelioma tumors when therapy was followed by a complete surgical removal [14]. These findings were reproduced by Grinshtein and colleagues who also demonstrated increased immunotherapy potency when combined with surgery in a neoadjuvant (pre-operative) setting [15]. Although these findings are very encouraging, the precise mechanisms underlying these observations have not been elucidated.

In this study, we further develop this concept of neoadjuvant immunotherapy followed by surgical cytoreduction. Two representative intratumoral immunotherapies currently in clinical trials were chosen to test the hypothesis. One approach utilizes a replication-defective adenoviral vector encoding for the HSV.*tk* protein (AdV-tk) in combination with ganciclovir (GCV). The HSV.*tk* gene monophosphorylates anti-herpetic prodrugs, such as GCV, that are further phosphorylated by endogenous cellular kinases into active triphosphate nucleotide analogs. These analogs are incorporated into cellular DNA, which results in an immunogenic cell death [16]. The second approach utilizes the intratumoral delivery of a replication-deficient adenoviral vector encoding for the type-I interferon, interferon-α (Ad.IFNα). Type-I interferons stimulate the immune system and have antitumor activity that includes immunoregulatory effects on antibody production, natural killer (NK) and T-cell activation, macrophage function, delayed-type hypersensitivity, and MHC antigen expression, in addition to anti-angiogenic properties and anti-proliferative effects [17]. We utilized two models of cytoreductive surgery which generate either local recurrences (partial tumor resection) or systemic recurrences (spontaneously metastatic cell lines with complete primary site resection).

We found that intratumoral immunotherapies are successful in treating limited disease by generating robust anti-tumor responses, but fail with increasing tumor burden despite generating anti-tumor immunocytes. Surgical cytoreduction restores the anti-tumor effects of immunotherapy by decreasing systemic MDSC populations, thus allowing enhanced CD8 T-cell trafficking and function. These data provide further support the paradigm of combining immunotherapy with surgical cytoreduction and provide one potential explanation for its additive effects.

## Results

### Intratumoral immunotherapy is effective in treating small tumors due to generation of anti-tumor CD8 T-cells

**Figure 1:**
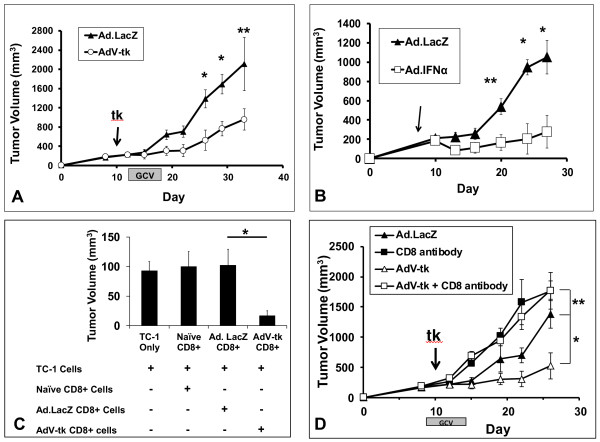
**Intratumoral immunotherapy is effective in treating early lung cancer due to enhanced CD8 T-cell function.** (**A**) AdV-tk/GCV (n = 8) or Ad.LacZ/GCV (control) (n = 8) was administered intratumorally at Day 11 when TC1 tumors measured ~250 mm^3^, and volume was recorded. AdV-tk/GCV provided a significant reduction in tumor volume. (**B**) Ad.IFNα (n = 8) or Ad.LacZ (control) (n = 8) was administered intratumorally at Day 8 when tumors measured ~250 mm^3^, and volume was recorded. Ad.IFNα provided a significant reduction in tumor volume. (**C**) CD8 T-cells form mice receiving AdV-tk/GCV were superior in neutralizing fresh tumor cells *in vivo* when compared to those CD8 T-cells harvested from mice receiving Ad.LacZ/GCV (n = 5 per group). (**D)** Mice (n = 8 per group) bearing early TC1 tumors were randomized to four treatment groups (*i*) AdV-tk/GCV, (*ii*) Ad.LacZ/GCV, (*iii*)AdV-tk/GCV and CD8 antibodies or (*iv*) Ad.LacZ/GCV and CD8 antibodies. Growth curves were plotted. Mice receiving CD8 antibodies, regardless of co-treatment, grew rapidly and with similar growth kinetics. * Represents a *p* < 0.05, ** represents a *p* < 0.01, shaded bar designates time over which AdV-tk/GCV therapy took place.

The anti-tumor effectiveness of intratumoral immunotherapy was first investigated using gene-mediated cytotoxic immunotherapy (GMCI) in early stage TC1 lung cancer flank tumors. Once tumors were established and measured ~250 mm^3^, animals were randomized to treatment with a single intratumoral injection of AdV-tk or Ad.LacZ. After 48 hours, both groups were treated with the prodrug GCV for five days. Mice randomized to AdV-tk/GCV were found to have significantly reduced tumor progression; p = 0.03 (Figure 1A). We similarly examined the anti-tumor effects of intratumoral cytokine immunotherapy as a treatment for early TC1 tumors using Ad.IFNα (or Ad.LacZ as control). Again, there were dramatic decreases in tumor volume in mice randomized to cytokine gene therapy; p = 0.009 (Figure 1A).

We next sought to determine if AdV-tk/GCV generates anti-tumor CD8 T-cells. To do this, we assayed splenic cytotoxic T lymphocytes using an *in vivo* tumor neutralization assay. To perform this assay, fresh TC1 tumor cells were mixed with CD8 T-cells isolated from the spleens of tumor-bearing mice treated with AdV-tk/GCV or Ad.LacZ/GCV; this mixture was then injected into tumor-naïve mice. When compared to controls, we observed significantly decreased tumor volume of tumors grown in the presence CD8 T-cells isolated from spleens of mice receiving GMCI; p = 0.01 (Figure 1C). To further demonstrate the key functional role of effector CD8 T-cells in AdV-tk/GCV treatment, CD8 T-cells were depleted with an anti-CD8 T-cell antibody prior to and during GMCI administration. Flow cytometric analysis confirmed CD8 T-cell depletion in our experimental groups ( Additional file 1: Figure S1). We found that selective elimination of CD8 T-cell populations negated the effects of GMCI protocols in animals bearing TC1 flank tumors; p = 0.006 (Figure 1D).

### Intratumoral immunotherapy has little effect in treating advanced tumor burden despite generating anti-tumor CD8 T-cell responses

**Figure 2:**
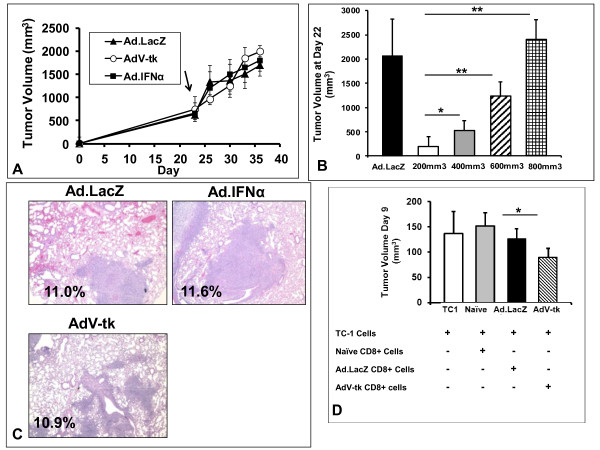
**Intratumoral immunotherapy is ineffective in the setting advanced tumor burden, and but produces functionally enhanced CD8 T-cells.** (**A**) C57BL/6 mice bearing locally advanced TC1 tumors (~750mm^3^) were randomized to treatment with AdV-tk/GCV, Ad.IFNα or Ad.LacZ/GCV (control) and tumor volume was recorded. No difference tumor growth was appreciated. (**B**) Mice (n = 5) bearing TC1 tumors of varying sizes (200mm^3^, 400mm^3^, 600 mm^3^ and 800mm^3^) were treated with AdV-tk/GCV. Mice (n = 5) bearing tumors measuring 200mm^3^ were treated with Ad.LacZ/GCV as a control. As can be seen, AdV-tk/GCV loses effectiveness as tumor burden increases as exemplified by tumor volume at Day 22. (**C**) Mice bearing LKR tumors, a spontaneously metastatic NSCLC line, were treated with AdV-tk/GCV, Ad.IFNα or Ad.LacZ/GCV (control) at a time point in which lung metastases are known to be present. Lungs were harvest at Day 42 and analyzed for tumor burden. Lung surface area involved with tumor burden was compared, and no difference was appreciated among groups. (**D**) *in vivo* tumor neutralization assay —Fresh TC1 tumor cells were mixed with (*i*) CD8 T-cells from mice receiving Ad.LacZ/GCV, (*ii*) CD8 T-cells form mice receiving AdV-tk/GCV and (*iii*) CD8 T-cells from tumor naïve mice and mixed into mice (n = 5). TC1 tumor cells were injected alone to confirm tumor cell viability. Trends toward enhanced CD8 T-cell function with intratumoral previous exposure were appreciated. * Represents a *p* < 0.05, ** represents a *p* < 0.01.

To more closely model advanced human cancers, we utilized two models of advanced disease: large established flank tumors (locally advanced disease) and spontaneously metastatic tumors (metastatic disease). To examine the anti-tumor potential of intratumoral immunotherapy in locally advanced disease, mice bearing large TC1 flank tumors (~700 mm^3^) were randomized to intratumoral inoculations of Ad.LacZ, Ad.IFNα or AdV-tk. After 24 hours, the mice treated with AdV-tk or Ad.LacZ were injected daily with GCV for 5 days. In contrast to the responses in small tumors, intratumoral immunotherapy had negligible inhibitory effects on tumor burden when administered in advanced tumors (Figure 2A).

To further explore the effectiveness of immunotherapy with regard to local tumor burden, we initiated AdV-tk/GCV therapy when tumors reached various sizes (200, 400, 600 and 800mm^3^) (Figure 2B). Although small (200 mm^3^) tumors were very susceptible to immunotherapy, anti-tumor effects of AdV-tk/GCV diminished as tumor size increased and eventually became completely ineffective. Similar relationships have been previously described when using type-I interferon cytokine gene therapy (Ad.IFNβ therapy) in treating TC1 tumors [14].

We then focused on the scenario modeling metastatic disease using the spontaneously metastatic NSCLC cancer line, LKR. We began by injecting LKR tumor cells into the flank of B6-129/J1 hybrid mice. When flank tumors reached the average size of 500 mm^3^ (Day 18), intratumoral immunotherapy (Ad.IFNα, AdV-tk/GCV, or Ad.LacZ/GCV) was begun. On day 42, after tumor cell inoculation, a time when lung metastases are known to exist, lungs were harvested, sectioned, and the surface area of the lungs and surface nodules measured. Again, we found no significant reduction in the percentage of lung surface area involved with tumor burden at Day 42; 11.6% (Ad.LacZ/GCV), 11.0% (AdV-tk/GCV), and 10.9% (Ad.IFNα); p = 0.76 (Figure 2C). Further, treatment did not reduce the number of lung metastases (data not shown).

Although no therapeutic benefit of AdV-tk and Ad.IFNα was appreciated in these advanced tumor settings, previous work with Ad.IFNβ has suggests that immunotherapy still generates CD8 T-cells capable of tumor neutralization [14]. With this concept in mind, we evaluated the ability of GMCI (AdV-tk/GCV) agents to generate cytotoxic CD8 T-cells in animals bearing large tumors. We again treated mice bearing locally advanced TC1 tumors (~700mm^3^) with Ad.LacZ/GCV or AdV-tk/GCV. Three days after GCV was completed, an *in vivo* tumor neutralization assay was performed by mixing TC1 tumor cells with CD8 T-cells isolated from the spleens of the aforementioned mice; this mixture was then injected into naïve mice. Interestingly, CD8 T-cells from animals that received AdV-tk*/*GCV therapy were more effective in inhibiting tumor growth than the other control groups; p = 0.03 (Figure 2D). This trend suggests that intratumoral immunotherapies are indeed able to generate anti-tumor CD8 T-cells, but these populations are inhibited by factors associated with advanced tumor states.

### Cytoreductive surgery restores the effectiveness of immunotherapy in advanced tumor states

**Figure 3:**
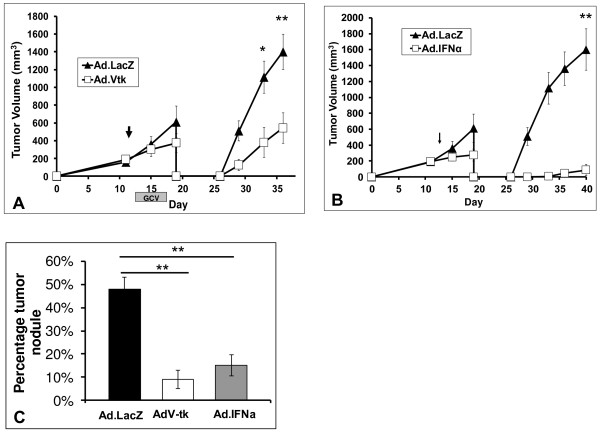
**Cytoreductive surgery restores effectiveness of immunotherapy even when administered in advanced tumor burden.** (**A**) Mice (n = 10) bearing large established tumors were randomized to AdV-tk/GCV or Ad.LacZ/GCV; two days following completion mice underwent positive margin surgery. Recurrent flank tumors were observed. We observed dramatic decreases in recurrent tumor volumes in those mice receiving neoadjuvant AdV-tk/GCV. (**B**) Similarly, mice (n = 10) bearing established tumors were randomized to Ad.IFNα or Ad.LacZ, followed by partial resection surgery 3 days later. Again, recurrent tumors were decreased in mice receiving intratumoral immunotherapy protocols. (**C**) We next tested this approach in the setting of metastatic disease. Mice (n = 14) bearing established LKR tumors were administered AdV-tk/GCV or Ad.LacZ/GCV at a time when metastatic lesions were known to be present. Two days after completion of drug administration, complete tumor excision was completed. On Day 40 of the experiment, lung tumor burden was assessed. Mice randomized immunotherapy had significantly less metastatic disease as compared to mice receiving control treatment. * Represents a *p* < 0.05, ** represents a *p* < 0.01.

Given that intratumoral immunotherapies generate CD8 T-cells, yet fail to elicit clinical responses in bulky disease, we aimed to investigate the concept of “adjuvant immunotherapy”. The concept is based on the premise that surgical resection can restore the immunobiology to that of a host with early disease—i.e. eliminate portions of immunosuppression that accompany advanced tumor states [18]. To test this hypothesis in locally advanced disease, we again treated mice bearing large flank tumors with either AdV-tk/GCV or Ad.LacZ/GCV. Following treatment, mice in both groups underwent *partial* resection to model cytoreduction [19]. Surgical sites were then monitored for recurrent disease. We found that mice treated with neoadjuvant AdV-tk/GCV had significantly decreased recurrent tumor burdens at all postoperative time points when compared to those mice randomized to control treatments; p = 0.006 (Figure 3A). We repeated this approach using cytokine gene therapy with Ad.IFNα and partial resection; again mice receiving neoadjuvant therapy had reduced postoperative volumes; p = 0.004 (Figure 3B).

This concept was next tested in the setting of advanced metastatic disease using LKR. Once flank tumors became established, mice were randomized to one of three treatment protocols: (1) AdV-tk/GCV, (2) Ad.IFNα, or (3) Ad.LacZ/GCV (control). Two days after treatments were completed, mice underwent *complete* flank tumor excision. Lungs were harvested on Day 40, and metastatic tumor burden was analyzed by assessing the percentage of lung surface area involved with tumor burden. On histologic analysis we found that those mice receiving immunotherapy treatments (AdV-tk/GCV or Ad.IFNα) had significant decreases in metastatic burden as compared to controls; p < 0.01 in both circumstances (Figure 3C).

Together these results suggest that intratumoral immunotherapy with cytoreduction surgery has the potential to be effective in treating advanced disease states.

### Intratumoral immunotherapy increases intratumoral trafficking and restores function of CD8 T-cells

**Figure 4:**
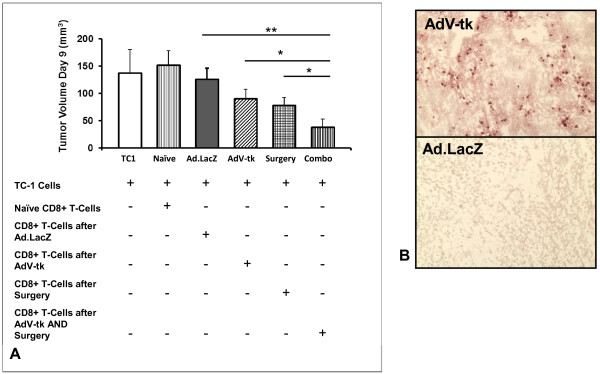
**Intratumoral immunotherapy prior to surgery is effective due to enhanced CD8 T-cell function.** (**A**) *in vivo* tumor neutralization assay. Mice bearing established flank tumors were randomized to one of four groups: (*i*) Ad.LacZ/GCV, (*ii*) AdV-tk/GCV, (*iii*) Ad.LacZ/GCV with cytoreduction surgery, and (*iv*) AdV-tk/GCV with cytoreduction surgery. CD8 T-cells were mixed with fresh tumor cells and injected into tumor naïve mice. After 9 days of tumor growth, we found that CD8 T-cells harvested from mice receiving AdV-tk/GCV and cytoreductive surgery inhibited tumor growth more potently than CD8 T-cells from other treatment groups. (**B**) Immunohistochemistry images of representative tumor from mice receiving AdV-tk/GCV or Ad.LacZ/GCV. Specimens were stained using anti-CD8 antibodies.

Our data had suggested that a central mechanism of GMCI lays in the generation of cytotoxic effector CD8 T-cell populations. To investigate the functional effects of combining cytoreductive surgery and immunotherapy in restoring CD8 T-cell anti-tumor activity, we again performed analyzed lymphocyte activity. In this scenario, mice bearing established flank tumors randomized to one of four groups: (1) Ad.LacZ/GCV, (2) AdV-tk/GCV, (3) Ad.LacZ/GCV with cytoreduction, and (4) AdV-tk/GCV with cytoreduction. CD8 T-cells were purified from splenocytes five days after the third and fourth groups underwent surgery, and were then mixed with fresh TC1 tumor cells. This mixture was then injected into the flanks of tumor naïve mice. After 9 days of tumor growth, we found that CD8 T-cells harvested from mice receiving *both* AdV-tk/GCV and cytoreductive surgery inhibited tumor growth more potently than CD8 T-cells from other treatment groups; p = 0.02 (Figure 4A).

To examine CD8 T-cell trafficking after surgery, we performed immunohistochemistry staining for CD8 T-cells in recurrent TC1 tumors of mice that were randomized to AdV-tk/GCV or Ad.LacZ/GCV (groups 3 and 4 from the previous experiment). Immunohistochemical staining demonstrated increased populations of CD8 T-cells in tumors of mice receiving AdV-tk/GCV compared to Ad.LacZ/GCV; 2.4% vs. 0.8% of total cells; p = 0.03 (Figure 4B).

This data demonstrates that the combination of immunotherapy and surgery have additive effects in augmenting effector CD8 T-cell population function and infiltration.

### Myeloid derived suppressor cells and immunosuppressive cyotkines are associated with advanced tumor burden

**Figure 5:**
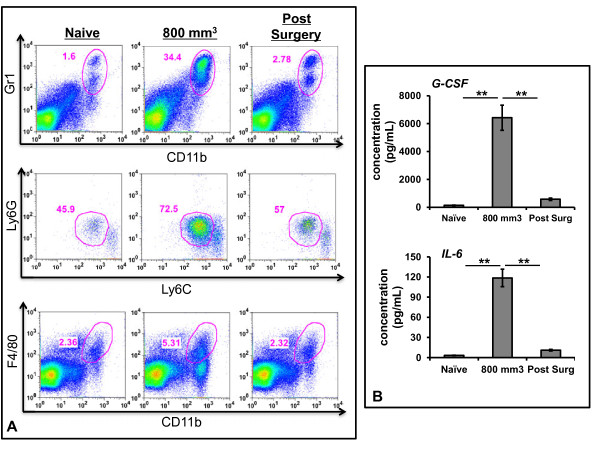
**Myeloid derived suppressor cells associated with advanced tumor burden are responsible for blunting immunotherapy potency.** (**A)** Flow cytometry of splenocyte populations obtained from (*i*) tumor naïve mice, (*ii*) mice bearing large, establish TC1 tumors or (*iii*) mice 3 days status post partial resection. ***Top-panel****—*Myeloid derived suppressor cells (CD11b+ Gr1+) were compared. MDSCs increased with tumor growth, but were found to be eliminated following cytoreduction surgery. ***Middle-panel—***Granulocytic MDSCs (Ly6G^hi^Ly6C^mod^) were also compared; and increased with tumor growth, but were found to be eliminated following cytoreduction surgery. ***Bottom-panel—***Splenic macrophages (Cd11b+F4/80+) increased with tumor growth were found to be eliminated following cytoreduction surgery. (**B**) Immunosuppressive cytokines (G-CSF and IL-6) in mouse serum at various times as measured by ELISA. Maximum levels were found in large tumors; surgical cytoreduction significantly decreased these cytokines to normal levels. * Represents a *p* < 0.05, ** represents a *p* < 0.01.

Advanced tumor states are associated with intricate immunosuppressive networks that subsequently impair anti-tumor effects of CD8 T-cells. Myeloid derived suppressor cells (MDSCs), which are defined as CD11b+Gr1+ cells in mice [18,20], are one such group. We were interested to see if cytoreduction decreased systemic MDSCs, as hypothesized by other groups [18,20]. To evaluate this concept, mice bearing large TC1 tumors were randomized to sham surgery or partial resection. At postoperative Day 3, mice were sacrificed and spleens were evaluated for MDSCs populations using flow cytometry; spleens from tumor naïve mice were also analyzed. We appreciated a significant increase in systemic MDSCs (as a fraction CD45 splenocytes) in mice bearing large tumors as compared to tumor-naïve mice; 4% to 18.6%; p < 0.01 (Figure 5A-top panel). Further, within three days of surgical cytoreduction, the percentage of MDSCs dramatically decreased to nearly normal level of 5.8%; p = 0.01 (Figure 5A top panel). Of note, there was an increase in the proportion of granulocytic MDSCs (Ly6G^hi^Ly6C^mod^ of total MDSCs) in mice bearing large tumors as compared to tumor naïve mice (44.6% vs. 71.3.7%; p = 0.01) (Figure 5A-middle panel). Following cytoreduction the percentage of granulocytic MDSCs decreased to near tumor-naïve levels (71.3% vs. 54.3%; p = 0.02) (Figure 5A-middle panel). Finally, we appreciated similar trends with regard to the percentage of macrophages (CD11b + F4/80+) of total splenic leukocytes: we observed an increase from 2.2% in tumor naïve mice to 5.4% in mice bearing large tumors (p = 0.02); following surgical resection the percentage of macrophages decreased to 2.3% (p = 0.02) (Figure 5A-bottom panel). These results suggest that reductions in immunosuppressive myeloid cells may be a mechanism by which CD8 T-cell function is restored following tumor cytoreduction.

We also focused on the serum presence of two immunosuppressive cytokines associated with MDSC generation and function (G-CSF and IL-6). Similar to cellular trends, mice bearing large primary tumors had significantly increased levels of both cytokines when compared to tumor naïve mice (Figure 5B). More specifically, we appreciated nearly a 60-fold increase of G-CSF (p < 0.001) and a 20-fold increase of IL-6 (p < 0.001). Following cytoreduction, serum levels of these immunosuppressive cytokines returned to baseline levels.

Together these finding demonstrate that advanced tumor states are associated with both increased cellular immunosuppressive populations, namely MDSCs, and elevated immunosuppressive cytokine levels. Further, surgical resection eliminates both of these factors.

### MDSCs are responsible for blunting the efficacy of intratumoral immunotherapy

**Figure 6:**
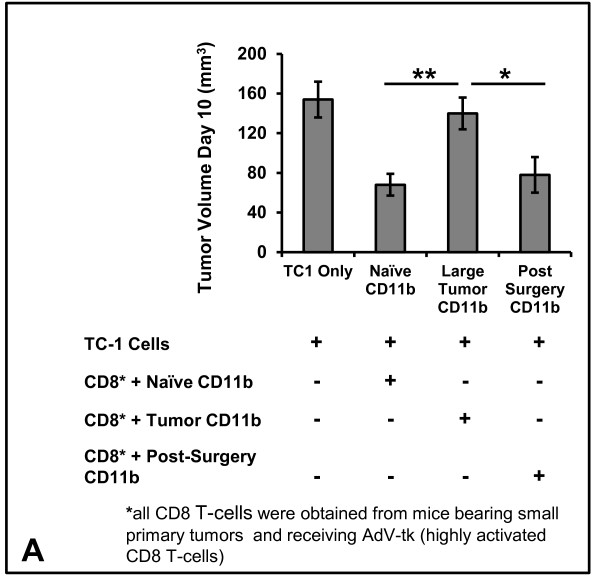
(**A**) **Mixing*in vivo*tumor neutralization assay**: Functionally active CD8 T-cells (obtained from spleens of mice bearing small primary tumors and after AdV-tk/GCV treatment) were mixed with fresh TC1 tumor cells and CD11b+ populations obtained at different points during progression: *(i)* tumor naïve mice, *(ii)* mice bearing large primary tumors or *(iii)* three days after surgical cytoreduction. Growth was then monitored for 10 days.* Represents a *p* < 0.05, ** represents a *p* < 0.01.

To confirm that MDSC populations inhibit activated CD8 T-cells in advanced tumor states, we performed a “mixing” *in vivo* tumor neutralization assay. Mice bearing small primary tumors were treated with AdV-tk/GCV (a time when GMCI generates CD8 T-cells with potent anti-tumor potential). From these mice, splenic CD8 T-cells were purified and mixed with fresh TC1 tumor cells and various CD11b populations (*i*. from tumor naïve mice, *ii*. from mice bearing large primary tumors or *iii*. mice which had undergone cytoreductive surgery). Cells were mixed at a tumor:CD8 T-cell:CD11b ratio of 1:3:10. These suspensions were injected into the flanks of tumor naïve mice and growth was monitored for 10 days. We noted that the presence of CD11b+cells isolated from mice bearing large tumors significantly blunted CD8 T-cell tumor neutralizing capabilities as compared to myeloid cells obtained from tumor naïve mice (p = 0.008) and mice undergoing surgery (p = 0.02) (Figure 6).

These findings provide *in vivo* evidence that increased MDSC populations associated with advanced tumor states inhibit CD8 T-cell neutralizing capabilities. Further, these findings support the hypothesis that surgery restores potency of CD8 T-cells via MDSC elimination.

## Discussion

The results of this study extend the concept of coupling cytoreductive surgery with neoadjuvant immunotherapy to restore the effectiveness of immunotherapy. To demonstrate this, we used two representative intratumoral immunotherapies which are currently being evaluated in clinical trials: an adenoviral vector encoding herpes simplex thymidine (AdV-tk) [1] and an adenoviral vector delivering the gene for interferon-α (Ad.IFNα) [2]. These immunotherapies have been effective in treating small tumors by CD8 T-cell dependent mechanisms. In contrast, as previous clinical data suggests, we found that immunotherapies had little anti-tumor effect in the setting of advanced disease. We demonstrate that this failure is not due to the inability of these therapies to generate CD8 T-cells, but the results of CD8 T-cell activity and trafficking. Although many mechanisms may result in impaired CD8 T-cell responses in large tumors (systemic immunosuppression, poor intratumoral vascularity, rapid tumor growth kinetics), we chose to focus on tumor associated immunosuppression (MDSCs).

We postulated that reducing the tumor burden would minimize the immunosuppressive networks, thus providing an opportunity for generated CD8 T-cells to neutralize tumor cells. We found that after cytoreducing large tumors, immunotherapies inhibited the rapid growth of both locally recurrent disease and systemic pulmonary disease. We further confirmed that these results were linked to CD8 T-cell mechanisms, namely increased trafficking and enhanced anti-tumor function. We appreciated that surgical cytoreduction significantly decreased the quantity of immunosuppressive MDSCs, a group cells associated with impaired adaptive immune responses [18,20].

In addition to supporting previous reports, this study expands on this the concept of a synergy between immunotherapy and surgery. First, we incorporate more accurate cancer surgery models in which disease predictably and spontaneously recurs locally or distantly. These approaches better mimic the human cancer scenario as compared to previous models that commonly include “rechallenging” with a second tumor cell inoculum following primary tumor resection [14]. Secondly, we begin to elucidate the mechanistic underpinnings which may explain the success of combining immunotherapy with surgical cytoreduction (i.e. loss of MDSC). Our data supports previous findings that immunosuppressive myeloid cells accumulate during tumor progression and contribute to immunosuppression by inhibiting the function of CD8 T-cells via immunosuppressive cytokine production (namely IL-6 and G-CSF), generation of oxygen radicals, and close cell-to-cell interactions [21]. It is likely that the CD8 T-cell generated by intratumoral immunotherapies are also subject to such effects. We have become encouraged by our findings and have developed a strong interest in pursuing a clinical trial which combines neoadjuvant immuno-gene therapy utilizing Ad.IFNα for malignant mesothelioma.

We demonstrate that surgery is a provocative mechanism to increase intratumoral immunotherapy potency. However, other approaches, including increasing vector dosage and frequency, are also reasonable solutions. Although feasible, previous evidence has shown only few cells actually need to be infected due to “bystander effects” [22], thus unnecessary dose escalation may also needlessly introduce harmful side-effect profiles.

There are important limitations to this report due to our syngeneic models of flank tumors. To date, xenograft models and orthotopic tumors originating from transgenic advances have emerged as gold-standard approaches to study cancer biology and therapeutics. The difficulty with such approaches in evaluating the potential synergy between immunotherapy and surgery is that xenograft models are established in immunodeficient hosts while orthotopic models typically arise in locations that are not amenable to surgical excision. Given these hurdles we believe that flank tumor approach at least provides a scenario approximating the clinical scenario. Further, we attempt to incorporate a model where metastatic recurrences are found in the lungs, thus mirroring the clinical scenario of distant metastasis after surgery.

## Conclusions

In summary, intratumoral immunotherapy is an appealing approach to treat cancers, however, to date it has had limited success in advanced disease states. Our study strengthens the concept of using *in situ* neoadjuvant immunotherapy approaches in patients with advanced tumor burden. The benefits of surgery and immunotherapy go beyond simple mechanical cytoreduction, and suggest a more complex interplay of immunological principles. We believe that with proper collaboration and further investigation, this multidisciplinary approach has the potential to have tremendous impacts that may be applicable to patients diagnosed with a variety of malignancies.

## Methods

### Animals

Female C57BL/6 mice (B6, Thy1.2) were purchased from Charles River Laboratories. Female C57BL/6J x 129P3/J hybrids (B6-129/J1) were purchased from The Jackson Laboratory. All mice were maintained in pathogen-free conditions and used for experiments at ages 8 week or older. Recognized principles of laboratory animal care (NIH publication No.85-23, revised 1985) were followed and the Animal Use Committees of the Children’s Hospital of Philadelphia, the Wistar Institute and the University of Pennsylvania approved all protocols.

### Cell lines

TC1 cells were derived from mouse lung epithelial cells of a C57BL/6 mouse, immortalized with human papillomavirus (HPV)-16 E6 and E7, and transformed with the c-Ha-ras oncogene {Lin, 1996 #1000}. The murine lung cancer line LKR was derived from an explant of a pulmonary tumor from an activated Kras G12D mutant mouse that had been induced in an F1 hybrid of 129Sv.J and C57BL/6 [23]. These cells were authenticated by morphologic, cell proliferation, and Mycoplasma tests, as recommended in ATCC Technical Bulletin No. 8 (2007).

TC1 cells were maintained in R10 media (RPMI supplemented with 10% heat-inactivated fetal bovine serum, 1% glutamine, and 1% penicillin and streptomycin (P/S)) and cultured at 37°C in a humidified incubator containing 5% CO_2_. LKR was maintained in D10 media (DMEM supplemented with 10% heat-inactivated fetal bovine serum, 1% glutamine, and 1% penicillin and streptomycin (P/S)) and cultured at 37°C in a humidified incubator containing 5% CO_2_.

### Vectors

The non-replicating serotype 5 adenovirus containing the herpes simplex virus thymidine kinase gene driven by a Rous sarcoma virus long terminal repeat promoter in the region of the deleted E1 wild-type adenoviral genes (AdV-tk) was produced by Advantagene. Control adenoviral vector contained the LacZ gene (Ad.LacZ) in the same position and was also produced by Advantagene**.** AdV-tk was produced in accordance with good manufacturing practices (21 CFR210 and 211). The vector has been characterized for purity and potency and approved for clinical use. The viral particle/infectious unit (IU) ratio was 10:1. Animals bearing TC1 tumors were treated with intratumoral (i.t.) injections of 1x10^10^ viral particles (vp) of AdV-tk or Ad.LacZ. After 48 hours, ganciclovir therapy was begun. The replication deficient serotype 5 adenovirus encoding a hybrid IFN-α2α1 (Ad.IFNα) with activity in mice was received from Schering-Plough, Inc. Mice were treated with one dose of Ad.IFNα i.t. at 1 x 10^9^ pfu. Vectors were used at this dosage in all experiments.

### Chemotherapy

Ganciclovir (GCV) was purchased from Roche Laboratories Inc. (Nutley, NJ) and was administered intraperitoneally at 50 mg/kg, once per day for five days, beginning at 2 days following intratumoral AdV-tk injection. GCV was dosed at 35 mg/kg post-operatively. GCV was suspended in 200μL normal saline for i.p. injections.

### Animal flank tumor models

Mice were injected subcutaneously (s.c.) on the right flank with 1 x 10^6^ of TC1 or 2x10^6^ LKR tumor cells in the appropriate mouse strain. 1.2x10^6^ of TC1 were injected s.c. into the flanks of syngeneic C57BL/6 mice. Mice were treated in one of four groups: (a) Control receiving normal saline or Ad.LacZ, (b) Normal saline or Ad.LacZ followed by surgery, (c) AdV-tk/GCV or Ad.IFNα, and (d) AdV-tk/GCV or Ad.IFNα. Surgery (described below) followed 3 to 5 after treatment completion. All experiments had at least five mice per group and were repeated at least once.

### Surgery

Two surgical models of cancer recurrence following surgery were utilized. Briefly, mice bearing flank tumors were anesthetized using intramuscular ketamine (80 mg/kg) and xyalazine (10 mg/kg) then shaved with hair clippers. A 1 to 2 cm incision was made immediately adjacent to the tumor. Resections were completed using standard blunt dissection. To produce a local recurrence, approximately 10% of the TC1 tumor burden was left at the tumor margin. Careful consideration was given to preserve blood supply to the remaining tumor volume. Occasionally it was necessary to remove skin that was adherent to the tumor. For the systemic recurrence model, the entire LKR flank tumor was completely resected (this was when pulmonary metastases were known to be present). Skin closure was performed using sterile silk 4-0 sutures. Buprenorphine (0.2 mg/kg) was administered at the time of surgery and 6 hours after as postoperative analgesia. Preoperative treatment was unknown to the investigator performing surgery and making tumor measurements. The Animal Use Committee of the University of Pennsylvania and the Wistar Institute approved all protocols in compliance with the Guide for the Care and Use of Laboratory Animals.

### Immunohistochemical studies

Mice were euthanized at the time tumors were harvested and frozen in Tissue-Tek OCT compound (Sakura Finetek USA, Inc., Torrance, CA) to be stored at −80°C. Five-micrometer sections were cut. Monoclonal antibodies against CD8 cells (anti-CD8) were obtained from BD Biosciences and immunohistochemical staining was performed according to established protocols. Tumor cell infiltrate was quantified by counting the number of positively staining cells in four high-powered (×400) fields. Five slides for each specimen were analyzed.

### Flow cytometric analysis

For flow cytometric analysis, tumors were removed from euthanized mice and minced into fine pieces in digestion buffer containing 0.1 mg/mL DNase I and 2.0 mg/mL collagenase type IV (Sigma, St. Louis, MO). Samples were incubated in digestion buffer at 37°C for 30 minutes, filtered through a 70-μm filter, and washed twice with R10. Fc receptors were blocked with anti-mouse CD16/CD32 antibodies (BD Biosciences PharMingen). Following one wash with PBS plus 2% FBS (staining buffer), cells were incubated for 30 minutes at 4°C with appropriate antibodies obtained from BD Biosciences PharMingen (San Diego, CA) and used at the indicated dilutions for flow cytometry: CD45-PerCP (1:200), CD8-APC (1:200), and CD11b-PerCP (1:200), Ly6G-PE (1:200), Ly6C-APC (1:200). Samples were then washed and resuspended in staining buffer or fixed in 2% paraformaldehyde. Splenocytes and lymph nodes were studied by FACS analysis. All fluorescently labeled antibodies used were purchased from BD Biosciences, except F4/80-APC and GR1-FITC (obtained from eBioscience). Flow cytometry was completed using a Becton Dickinson FACS Calibur flow cytometer (San Jose, CA), and analyzed using FlowJo software (Ashland, OR).

### Functional in vivo lymphocyte killing assay

To determine the amount of suppressive activity found in splenocytes from animals bearing large tumors, we used a modification of an *in vivo* tumor neutralization assay [24]. Briefly, CD8 T-cells or CD11cells were isolated from the spleens of mice using the MACS system (Miltenyi Biotec, Auburn, CA). These CD8 T lymphocytes were mixed with viable TC1 tumor cells in ratios of three purified CD8 splenocytes to each tumor cell (3:1). The mixture (containing 0.5 × 10^6^ TC1 cells and 1.5 × 10^6^ CD8 T-cells) was inoculated into the flanks of naïve mice. This ratio of CD8 T-cells and tumor cells has previously been determined to be optimal for detecting positive and negative effects [25]. When CD11b cells were used in a “mixing *in vivo* tumor neutralization assay”, a tumor:CD8 T-cell:CD11b ratio of 1:3:10 was utilized. Tumor growth was measured for 8 to 10 days and expressed as the mean ± SE of at least five mice per group.

### Statistical analyses

For flow cytometry, immunohistochemistry, real time RT-PCR and flank tumor volume studies comparing differences between two groups, we used unpaired Student’s t tests. For studies comparing more than two groups, ANOVA with appropriate post hoc testing (Bonferroni) was implemented. Differences were considered significant when p < 0.05. Data are presented as mean (standard error), unless otherwise noted.

## Abbreviations

TGF-β, Transforming Growth Factor-Beta; MDSC, Myeloid Derived Suppressor Cell; IFN, Interferon; GCV, Ganciclovir; GMCI, Gene-mediated cytotoxic immunotherapy.

## Competing interests

The authors declare that they have no competing interests.

## Authors’ contributions

JP assisted in study design, data collection, data analysis, and manuscript preparation. VK assisted in study design, data collection and data analysis. BJ assisted in data collection and data analysis. GC assisted in data collection and data analysis. ZF assisted in study design, data analysis and manuscript preparation. SA assisted in study design, data collection, data analysis and manuscript preparation. SS assisted in study design, data collection, data analysis and manuscript preparation. All authors read and approved the final manuscript.

## Supplementary Material

Additional file 1 **Figure S1.** Flow cytometry tracing confirming depletion of CD8 T-Cells from splenocyte populations in mice receiving CD8 antibodies. Click here for file
