# Supplementary figures and images for: Cytoreduction surgery reduces systemic myeloid suppressor cell populations and restores intratumoral immunotherapy effectiveness
Source: J Hematol Oncol. 2012 Jun 28;5:34. doi: 10.1186/1756-8722-5-34 (PMC3418164; doi:10.1186/1756-8722-5-34)

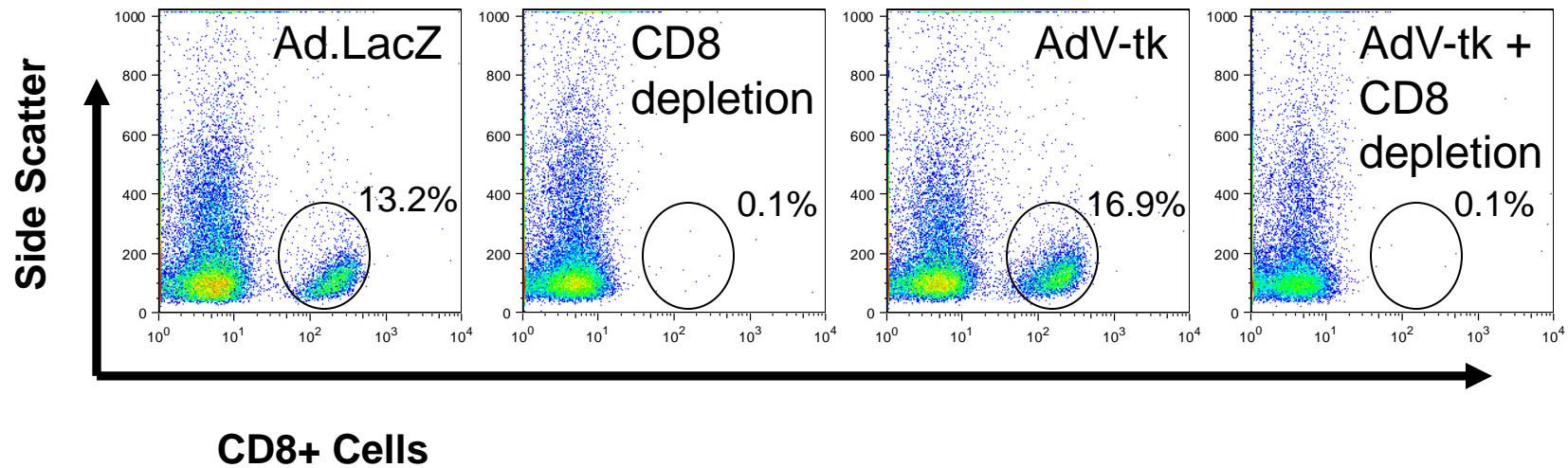

**Supplemental Figure 1**

Supplement: Additional file 1 — Figure S1. Flow cytometry tracing confirming depletion of CD8 T-Cells from splenocyte populations in mice receiving CD8 antibodies. [file 1756-8722-5-34-S1.pdf]
